# Diagnosis and Treatment of a Morel-Lavallee Lesion via Point of Care Ultrasound (POCUS)

**DOI:** 10.24908/pocus.v9i2.17699

**Published:** 2024-11-15

**Authors:** Djoser Mack, Joselyn Miller, Frank Yuan, Edison Lee, Trent She

**Affiliations:** 1 Department of Emergency Medicine, University of Connecticut Farmington, CT USA; 2 Department of Orthopedic Surgery, Hartford Hospital Hartford, CT USA; 3 Department of Radiology, Hartford Hospital Hartford, CT USA; 4 Department of Emergency Medicine, Hartford Hospital Hartford, CT USA

**Keywords:** Morel-Lavallee Lesion, Clinician-Performed Ultrasound, Case Report, POCUS

## Abstract

Morel-Lavallee Lesion (MLL) is a rare diagnosis of a closed internal degloving injury that can occur with high energy trauma. The pain, soft tissue swelling, and ecchymosis that patients describe mimic many other emergent diagnoses to include compartment syndrome and fractures. The following case highlights the importance of the role of Emergency Medicine physicians using point-of-care ultrasound (POCUS) to recognize and treat a potentially life-threatening injury. Our patient was initially managed with a bedside needle aspiration with drainage of 25cc of serosanguinous fluid that resulted in immediate pain relief. Patient was then admitted for further Interventional Radiology drainage of 160cc of serosanguinous fluid by Interventional Radiology.

## Case Presentation

**Figure 1:**
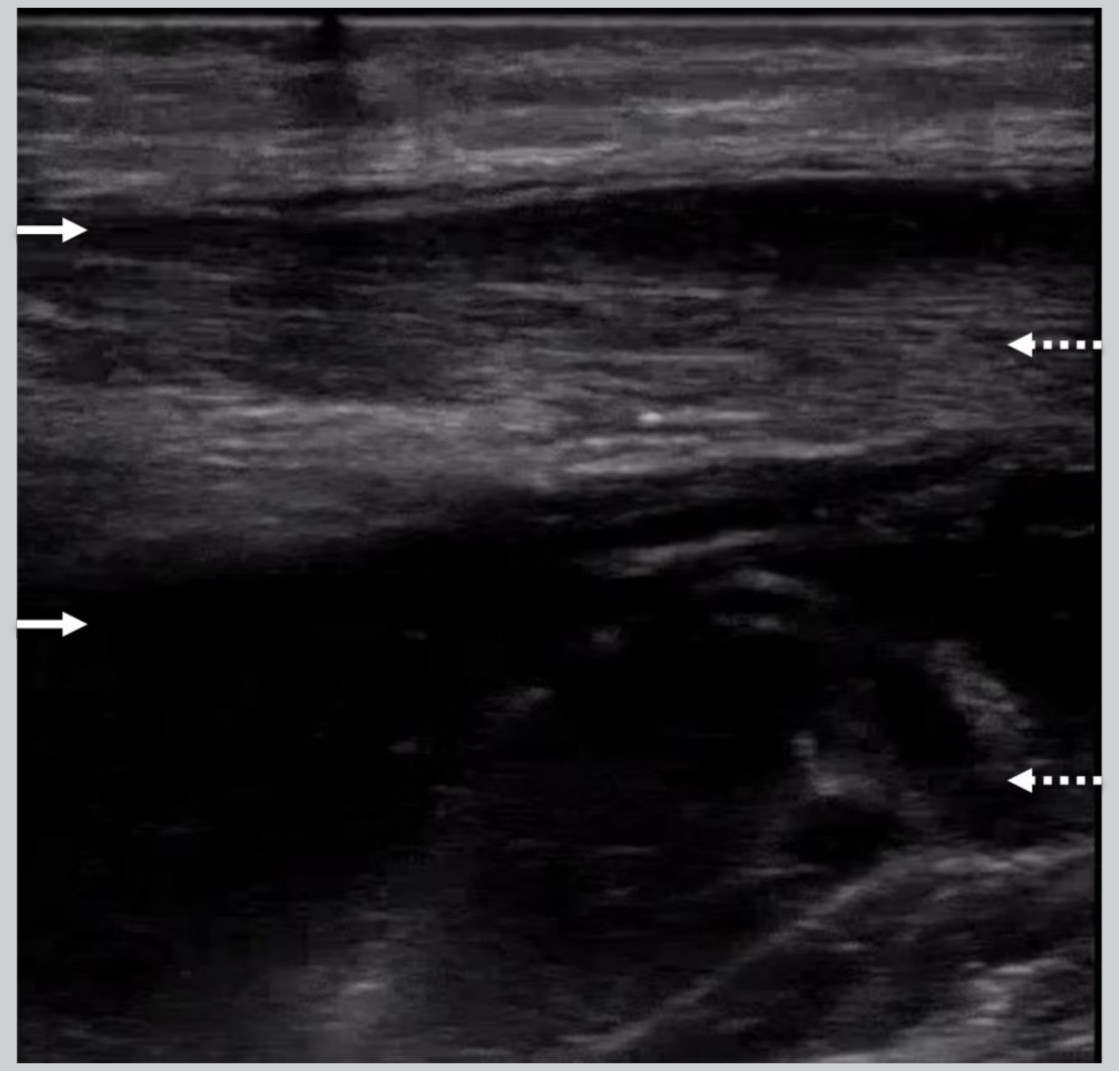
POCUS image of the anteromedial left upper arm in sagittal position with the high frequency linear transducer in the emergency department on day 1.Two hypoechoic pockets of fluid are noted traveling distally in fascial planes (two solid white arrows) between the two heads of the biceps muscle (two dashed white arrows).

**Figure 2:**
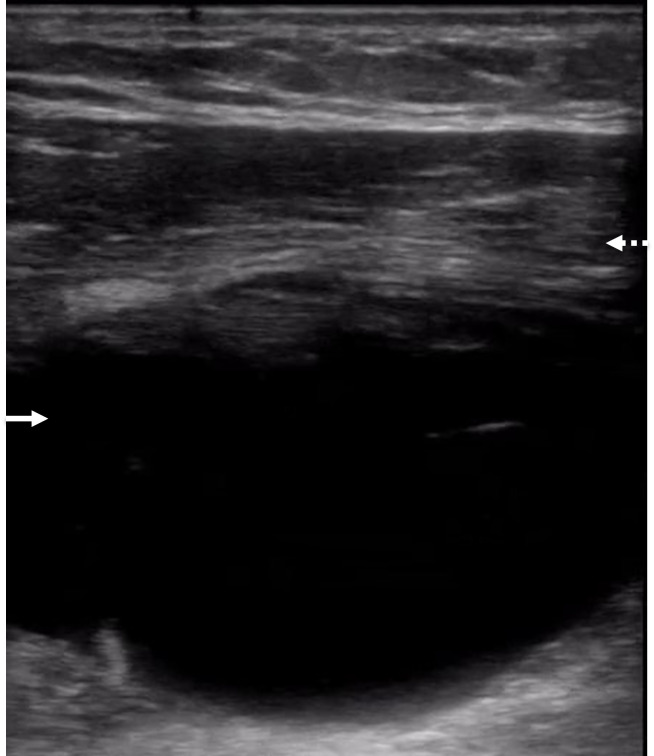
Point of care ultrasound image of the anterolateral chest in axial position with the high frequency linear transducer in the emergency department on day one. A large fluid collection (solid white arrow) is noted to significantly deform the underlying musculature while traveling along the underside of the pectoralis major muscle (dashed white arrow).

**Figure 3:**
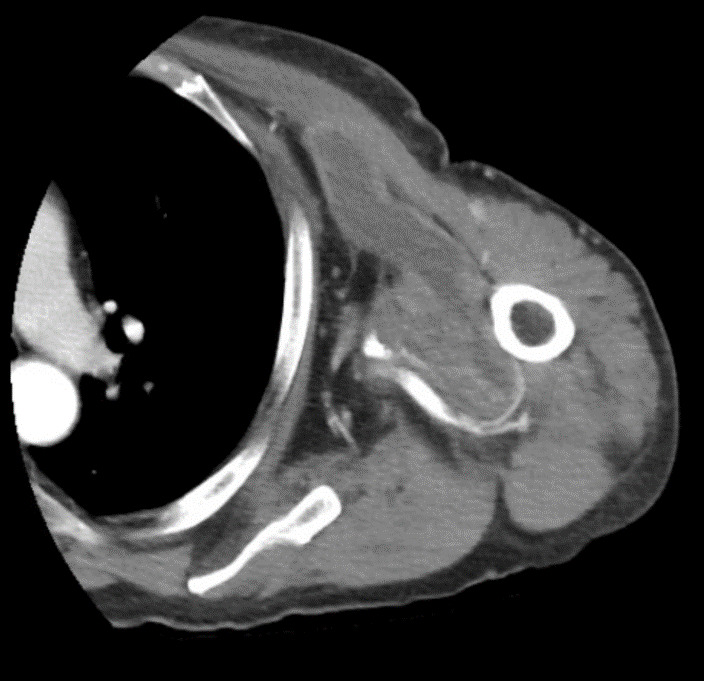
Computed tomography scan showing fluid collection (solid white arrow) traveling from the chest to the upper arm between the pectoralis and biceps muscle regions.

**Figure 4:**
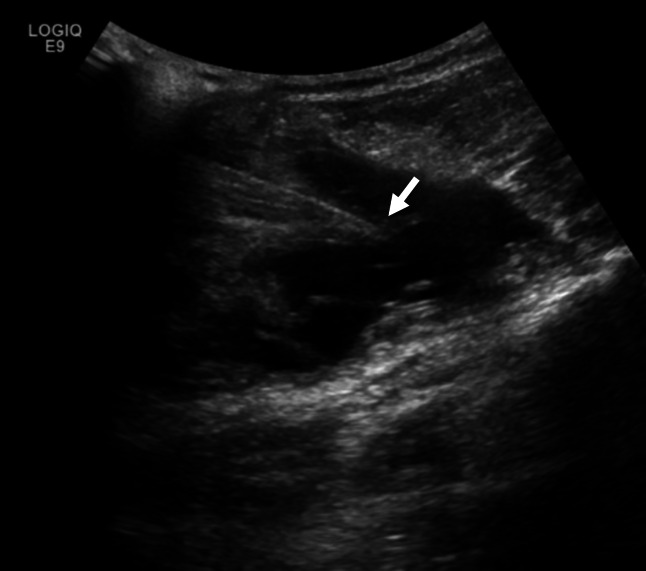
Interventional radiology ultrasound guided drainage of the Morel-Lavallée Lesion with the curvilinear probe on day two. The needle is noted to just penetrate the fluid pocket (solid white arrow).

**Figure 5:**
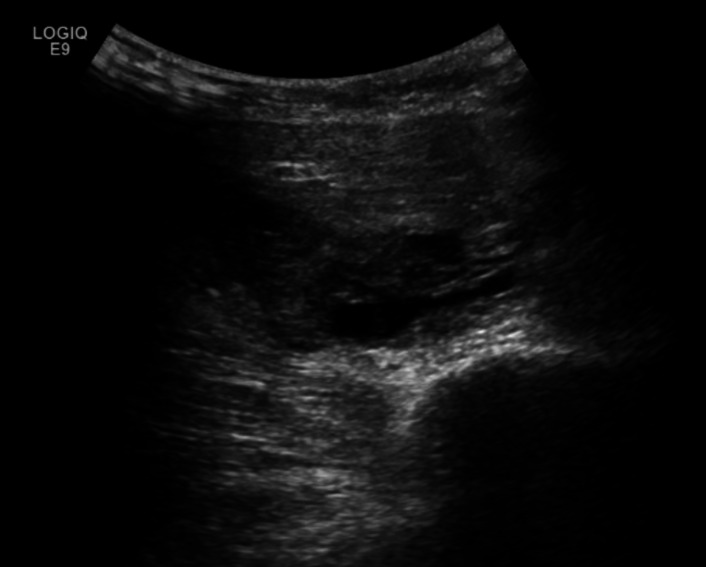
Post procedural ultrasound image showing a significantly improved fluid collection compared to Figure 4.

A 68-year-old male presented to the Emergency Department (ED) with worsening left upper extremity (LUE) pain and swelling after a fall on his outstretched arm one week ago. On physical examination, he had intractable LUE pain, numbness, limited range of motion, bruising, and a tense anterior compartment of the arm concerning for compartment syndrome. Point of care ultrasound (POCUS) demonstrated an extensive fluid collection tracking from the pectoralis muscle to the upper LUE suggestive of a Morel-Lavallée Lesion (MLL) (Figures 1, 2). Computed tomography (CT) confirmed the diagnosis (Figure 3). ED providers drained 25cc of fluid via POCUS-guided needle aspiration allowing for some relief of his symptoms. The patient was admitted and had 160cc of serosanguinous fluid drained by interventional radiology (Figures 4, 5). 

MLLs are a result of a high-energy traumatic shearing injury that lead to internal degloving and separation of fascial layers 1. This leaves a potential space for blood, lymph and serous fluid to accumulate which can lead to tissue necrosis if not promptly identified 2, 3. Classically, the diagnosis of MLL was made with CT or Magnetic Resonance; however, with POCUS, providers can both rapidly diagnose, as well as treat, patients at bedside. Under POCUS, MLLs appear as fluid collections tracking between fascial layers with fat globules and septations 4. Although MLLs usually present on lower extremities, this report describes an upper extremity lesion found through POCUS and CT. Standardizing the use of POCUS in scenarios where MLLs are suspected is beneficial for early recognition and intervention, thus decreasing complications of MLLs.

## Informed Consent Statement

This case report includes images obtained during the patient encounter. We have formally requested and received consent from the patient regarding use of these de-identified images and videos for submission.

## Disclosure Statement 

The authors declare that they have no competing interests.

## Supplementary Material

• Video S1

• Video S2

• Video S3

• Video S4
